# Egocentric Distance Perception Disorder in Amblyopia

**DOI:** 10.5334/pb.1038

**Published:** 2021-06-21

**Authors:** Bo Dong, Airui Chen, Tianyang Zhang, Ming Zhang

**Affiliations:** 1 Department of Psychology, Suzhou University of Science and Technology, Suzhou, China; 2 School of Public Health, Soochow University, Suzhou, China; 3 Department of Psychology, Soochow University, Suzhou, China

**Keywords:** egocentric distance perception, amblyopia, personal space, action space, vista space

## Abstract

Egocentric distance perception is a psychological process in which observers use various depth cues to estimate the distance between a target and themselves. The impairment of basic visual function and treatment of amblyopia have been well documented. However, the disorder of egocentric distance perception of amblyopes is poorly understood. In this review, we describe the cognitive mechanism of egocentric distance perception, and then, we focus on empirical evidence for disorders in egocentric distance perception for amblyopes in the whole visual space. In the personal space (within 2 m), it is difficult for amblyopes to show normal hand-eye coordination; in the action space (within 2 m~30 m), amblyopes cannot accurately judge the distance of a target suspended in the air. Few studies have focused on the performance of amblyopes in the vista space (more than 30 m). Finally, five critical topics for future research are discussed: 1) it is necessary to systematically explore the mechanism of egocentric distance perception in all three spaces; 2) the laws of egocentric distance perception in moving objects for amblyopes should be explored; and 3) the comparison of three subtypes of amblyopia is still insufficient; 4) study the perception of distance under another theoretical framework; 5) explore the mechanisms of amblyopia by Virtual Reality.

## 1 Introduction

*“Considering the tremendous scientific effort expended on understanding amblyopia and its clinical management, the lack of research on its functional impact is simply stunning and is a sad reflection of the level of interaction between basic and clinical science.”*

*Fielder, 2002*

Vision is essential to human beings, and our perception is often dominated by vision. Healthy people may be accustomed to this seemingly innate ability. However, people suffering from eye diseases have to strive to read, study and work. Impaired vision makes a lot of troubles during the individual’s lifetime. Disorders or diseases for eyes are quite common. People whose life is relatively longer may suffer from one kind of eye disease or more during their lifetime. According to a WHO (World Health Organization) survey, there are at least 2.2 billion people with impaired vision or blindness, of whom at least 1 billion have vision impairment that could have been prevented or has yet to be addressed (WHO, 2019). Amblyopia is weakened visual ability caused by a lack of experiences from visual modality during the initial stage of people’s lives (Birch, 2013; Levi et al., 2015). Clinically, amblyopia is weakened visual acuity with strabismus, anisometropia, high refractive error, or/and cataracts. At the critical stage of maturity, amblyogenic factors play a negative role in the development of the visual cortex. Thus, humans suffer from amblyopia and have impaired visual function.

Amblyopia is a common visual disease. The global average prevalence of amblyopia is approximately 1.75%, with regional and age differences (Hashemi et al., 2018). Specifically, the highest prevalence of amblyopia was found in Europe (approximately 3.67%), and the lowest prevalence was found in Africa (approximately 0.51%); incidence in the United States was approximately 2.77%, in Western Pacific countries was approximately 1.19% (Hashemi et al., 2018), and in China was approximately 1.2% to 3.53% (Hu et al., 2016). In China, for example, the prevalence in preschool children aged 3 to 6 years was 1.2% to 1.47% (X. Chen et al., 2016; Huang et al., 2018), that of school-age children aged 6 to 14 years was 3.53% (Pan et al., 2017), that of junior high school students aged 10 to 16 years was 2.5% (Fu et al., 2014), and that of people aged 30 to 80 years was 2.8% (Y. Wang et al., 2011).

According to the predominant theory of amblyopia, the mismatch between the retina images of two eyes leads to the preference for information in one eye and the suppression of the other eye, resulting in neurological disorders in the visual cortex; thus, people ultimately suffer from amblyopia (Birch, 2013; Harrad et al., 1996; Hess et al., 2014). Defects in basic visual function and treatments for amblyopia have been well documented (Bao et al., 2018; Dong et al., 2014; J. Zhou et al., 2015). However, the functional disorders for everyday activities in amblyopes’ daily life are poorly understood (Fielder, 2002; Grant & Moseley, 2011). In fact, the quality of life is significantly lower for amblyopes than for healthy people. Amblyopes not only face visual blur, decline of distance judgement, diplopia (i.e., double vision, which is when you see two images of the same thing) and other functional disorders but also bear psychological distress, such as a lack of self-confidence, depression, and family tension (Carlton & Kaltenthaler, 2011; Holmes et al., 2003; Koc et al., 2013; Z. Wang et al., 2014).

Egocentric distance perception is a psychological process in which observers use various depth cues to estimate the distance between the target and themselves (Blake & Sekuler, 2006; Howard, 2012). It plays an important role in amblyopes’ quality of life. Approximately 40% of the items tested on the main quality of life scales (e.g., the Amblyopia and Strabismus Questionnaire, ASQE; 20-item Adult Strabismus questionnaire, AS-20; the Amblyopia Treatment Index, ATI) are related to egocentric distance perception (Cole et al., 2001; Hatt et al., 2009, 2010; Van De Graaf et al., 2004). These items include “I can estimate the distance well”, “I can’t park the car in the exact position”, and “I feel difficulty when I go down the stairs”, and so on. These feelings and experiences indicate disorder in egocentric distance perception. The disorder of egocentric distance perception was proven by these subjective experiences (Carlton & Kaltenthaler, 2011; Y. Chen et al., 2016; Koc et al., 2013; Vianya-Estopa et al., 2010; Z. Wang et al., 2014). Nevertheless, the results of scales may be affected by individual emotions and/or judgement criteria of children or their parents, and it is difficult to objectively measure egocentric distance perception. In this article, we introduce the cognitive mechanism of egocentric distance perception at first, and then, we focus on experimental evidence rather than questionnaire surveys in relation to amblyopes’ egocentric distance perception disorders. Specifically, we clarify the behavioural performance of amblyopes in personal space (within 2 m) and action space (within 2 m~30 m). Finally, five critical scientific questions regarding investigating amblyopia are discussed.

## 2 Cognitive Mechanism of Egocentric Distance Perception

Locating the target and judging its distance are vital for human survival (Proffitt, 2006a). For instance, pedestrians always judge the distance between themselves and other pedestrians when walking; drivers judge the distance from other vehicles when driving; and pilots of carrier aircraft perceive the distance and direction of aircraft carriers when landing. In these tasks, the participant generally uses internal and external information to locate the target. In our daily life, these two kinds of depth cues mainly exist in the ground-surface reference system.

### 2.1 Utilization of external depth cues

**Figure 1:**
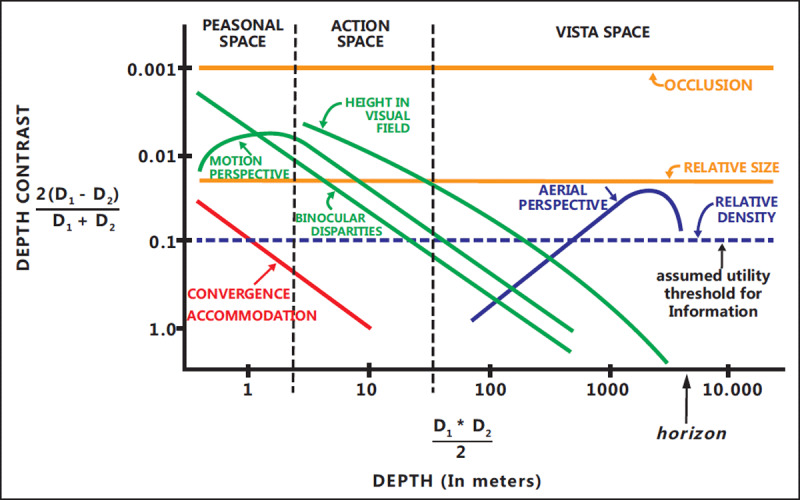
**Depth contrast sensitivity of cues in personal, action and vista spaces**. The horizontal axis represents distance, and the distance gradually increases from left to right. The vertical axis represents the discrimination threshold of depth, which means the shortest magnitude at which observers could distinguish two distances. The smaller the vertical axis value is, the more sensitive the observers. As we can see from the figure, the strength of binocular convergence and the sensitivity of lens accommodation are pretty high in the personal space; observers could sensitively perceive the cues provided by binocular disparities, motion perspective and relative height in the action space. In the vista space, area perspective and relative density cues play a key role. In all three spaces, the sensitivity to obstacles and relative size cues remained higher than the average (Cutting & Vishton, 1995).

Cutting (1995) divides the whole space around us into three parts according to the egocentric distance (Cutting & Vishton, 1995). First, the space that people can explore through their hands (within 2 m from observers) is called personal space. Humans can precisely operate objects in this space with their hands. Second, the area beyond arm length and within 30 m is the action space. The main activities of human beings are conducted in this space. In the space, human beings can move quickly, socialize with others, and throw things at their enemies or to their teammates. Third, the area within the field of vision but beyond the action space is called the vista space. Objects or events in this space are useful for navigation, approach and escape. In addition to the differences in the way people interact with the environment, there are also differences in the information that people use to judge egocentric distance in the three spaces (Cutting & Vishton, 1995). In personal space, people generally judge distance based on binocular convergence and accommodation of the lens. Binocular disparities, motion perspective and relative height are commonly used as depth cues in the range of action space. In the vista space, area perspective and relative density become the main cues. The occlusion and relative size are the depth cues used in all three spaces (***Figure 1***). The efficiency of using these depth cues directly affects the accuracy of distance perception. It is noteworthy that depth cues often appear in the form of combinations in the real world.

**Figure 2:**
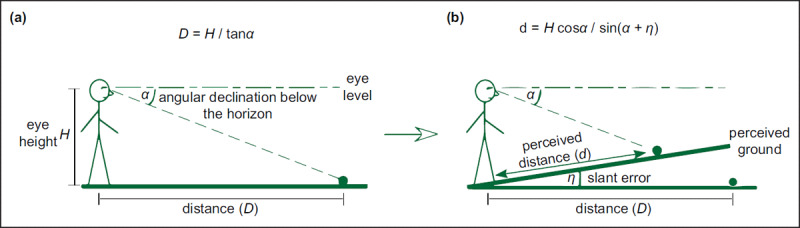
**The formula for egocentric distance perception. (a)** The distance between the target and observer can be calculated by the eye height (*H*) and the angular declination (*α*), that is, *D = H/tan(α)*. **(b)** the visual system perceives the ground as an upwardly tilted surface in the distance (black slant line), and the slope *η* represents the angle between the tilted surface and the horizontal plane (grey horizontal line). Therefore, the egocentric distance *D* is determined by *H, α* and the slope *η*, that is, *D = H cos α/sin (α + η)* (Ooi & He, 2007).

The ground surface plays an important role in distance perception (Gibson, 1950; Ooi & He, 2007). The distance D between the target and the observer can be calculated by the eye height (*H*) and the angular declination (*α*), that is, *D* = *H*/tan *α* (***Figure 2a***). There is an important premise for the establishment of this formula: the visual system accurately perceives the ground as a horizontal plane and can accurately characterize *H* and *α* simultaneously. In fact, however, the visual system of people (amblyope or not) can only accurately represent *H* and *α* (Ooi et al., 2001, 2006; J. Wu et al., 2005) and inaccurately perceive the ground as a bevelled upwards surface in the distance (He et al., 2004; Ooi et al., 2001, 2006). Therefore, there is an underestimation of the actual distance, resulting in compression of space (Gilinsky, 1951; Ooi & He, 2007). Consequently, the egocentric distance *d* is determined by *H, α* and the slope *η* of the ground surface, specifically, *d* = *H* cos *α*/sin (*α* + *η*) (***Figure 2b***). Gibson (1950) also clarified the important role of the ground in distance perception and proposed the ground theory of space perception (Gibson, 1950, 1979), believing that the size of *η* would be affected by depth cues on the ground. Studies have shown that when the ground is a continuous uniform surface, the judgement of distance is relatively accurate, while the judgement becomes inaccurate when there are obstacles, ditches in the ground or a sudden change in the texture gradient (Dong et al., 2017; He et al., 2004; Sinai et al., 1998; B. Wu et al., 2004, 2007).

### 2.2 Utilization of intrinsic bias

**Figure 3:**
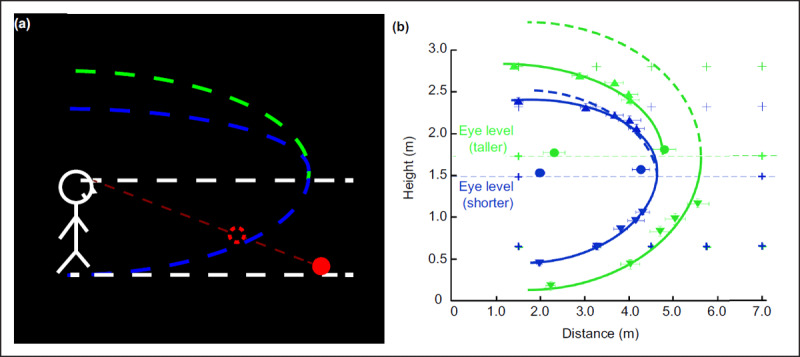
**Schematic of intrinsic bias. (a)** The intrinsic bias in three-dimensional space. Intrinsic bias is an asymmetric conicoid-like surface, including the lower and upper parts of intrinsic bias divided by the eye height level. The upper part with higher curvature is closer to the top of the participant’s head than the lower part is. In a dark environment, observers take the intrinsic bias as a frame of reference for space perception and locate the object at the intersection of line of sight and the intrinsic bias surface. **(b)** The effects of observer height on intrinsic bias. The blue line represents the intrinsic bias of short observers, while the green line represents the intrinsic bias of the tall observers. The solid line above is the actual intrinsic bias curve, and the dotted line is the curve symmetric with the intrinsic bias below. Therefore, for the taller, the curvature of the lower part is lower and the curvature above is higher than those of shorter. Reprinted/adapted from (L. Zhou, Ooi, & He, 2016). The Authors, some rights reserved; exclusive licensee AAAS. Distributed under a Creative Commons Attribution NonCommercial License 4.0 (CC BY-NC) *http://creativecommons.org/licenses/by-nc/4.0/*.

In addition to the external cues mentioned above, *η* is also affected by the internal representation of the visual system, i.e., intrinsic bias (Ooi et al., 2001, 2006; L. Zhou et al., 2016, 2017). Intrinsic bias is an asymmetric conicoid-like surface, which is a highly internalized and stable representation of internal space based on personal life experiences. It includes the lower and upper parts of intrinsic bias divided by the eye height level. The lower part of the intrinsic bias is a recessive surface with an upward inclination angle of 10~13° from the ground. The upper part is similar but has a higher curvature, and it is closer to the top of the participant’s head (***Figure 3a***). Moreover, whether in an environment with numerous cues (such as a well-illuminated playground), rare cues (such as a dark corridor) or a fully dark environment, intrinsic bias may affect distance perception in a certain way (Ooi et al., 2006; L. Zhou et al., 2013). For example, in a dark environment, an individual takes the intrinsic bias as a frame of reference for space perception and locates the object at the intersection of line of sight and the intrinsic bias surface (***Figure 3a***). There are differences between individuals’ intrinsic biases. For example, the curvature of the lower part is lower and the curvature above is higher for taller participants than for shorter participants (***Figure 3b***) (L. Zhou et al., 2016). According to the formula *d* = *H* cos *α*/sin (*α* + *η*), people with higher eye height perceive a greater distance. Over time, taller individuals can be accustomed to perceive farther (i.e., the green line on the periphery of the blue line in ***Figure 3b***).

In summary, egocentric distance perception is a process in which an observer perceives the location of a target by using external depth cues and internal intrinsic bias, which is very important in daily life. Egocentric distance perception could be affected by a variety of external cues, the sensitivity of individuals to cues, and individual differences in intrinsic bias. Therefore, if some diseases make patients insensitive to one or more kinds of depth cues (i.e., inability to perceive binocular disparity cues and texture with high density), it may affect egocentric distance perception, which may be reflected in the utilization of external cues and the existing intrinsic bias. Amblyopia is one such disease, and amblyopes may have many impairments in visual function, including visual acuity impairment in deterioration, contrast sensitivity reduction and stereo vision loss. (McKee et al., 2003). These impairments not only make it difficult for patients to perceive image details and observe the stereoscopic effect of 3D movies but also reduce the accuracy of distance perception and affect their quality of life.

## 3 Egocentric Distance Perception Disorder in Amblyopes

Egocentric distance perception disorder in amblyopes may be observed in the whole space. In the personal space, it is difficult for patients to show normal hand-eye coordination (Grant & Moseley, 2011); in the action space, patients cannot accurately judge the distance of a target suspended in the air (Ooi & He, 2015). Few studies focus on the performance of amblyopes in the vista space. Depth cues for distance judgement in the three spaces are different (Cutting & Vishton, 1995); amblyopes may therefore have different pathogenesis mechanisms of egocentric distance perception disorder in personal, action and vista spaces.

### 3.1 Egocentric distance perception disorder in personal space

In personal space, the disorder in egocentric distance perception for amblyopes is that the hand-eye coordination ability of amblyopes is lower than that of healthy people. Hand-eye coordination refers to the process in which observers use visual input and proprioception to guide hand movement while touching, grasping and manipulating objects. It is of great importance for daily activities (such as reading, fetching, and movement) (Grant & Moseley, 2011; Melmoth & Grant, 2006). The researchers measured manipulation, velocity, and sensitivity for amblyopes at preschool age (3-5 years old), middle school age (6-10 years old) and after school age (10-30 years old) using a bead-string task, nail-pulling task and painting task, respectively. The results showed that the performance of amblyopes in most tasks was obviously inferior to that of normal participants, and such dyskinesia was particularly evident in tasks with limited time in a 3D environment (Hrisos et al., 2006; O’Connor et al., 2010; Verghese et al., 2016; Webber et al., 2008). Whether in the condition of binocular, strong-eyed viewing or amblyopic eye viewing, amblyopes showed disorders in action preparation, action execution (speed of movement) and action performance (accuracy). In the phase of action execution, there were obviously prolonged preparation stages and acceleration stages, as well as a decreased maximum speed in amblyopes (Niechwiej-Szwedo, Goltz, Chandrakumar, Hirji, & Wong, 2011; Niechwiej-Szwedo, Goltz, Chandrakumar, Hirji, Douglas Crawford, et al., 2011). Amblyopic children spent twice as much time touching the target, and the failure probability of hand movement direction and position was 1.5 to 3 times that of normal children (Suttle et al., 2011). Furthermore, amblyopic adults also have hand-eye coordination disorders. The difference between amblyopic children and adults is that amblyopic adults move faster than amblyopic children, and adults do not show significant deficiencies under the condition of strong-eyed viewing (Grant et al., 2007; Niechwiej-Szwedo, Goltz, Chandrakumar, Hirji, Douglas Crawford, et al., 2011). In other words, amblyopic adults are the same as normal adults under the condition of single-healthy eye viewing. Amblyopic adults have disorders in action planning and execution only, which are manifested by longer action planning time, longer acceleration time and slower maximum speed (Niechwiej-Szwedo et al., 2014). When the target is surrounded by other objects, the hand-eye coordination deficit is more obvious in amblyopes (Buckley et al., 2015). In addition, amblyopic convalescents who have recovered their vision ability still suffer from stereo-acuity impairment and show hand-eye movement defects similar to those of amblyopic people (Melmoth et al., 2009). Reducing ambient brightness or five hours of blurred adaptation has no significant impact on hand-eye coordination; however, reducing target contrast aggravates hand-eye coordination disorder in amblyopic patients (Grant & Conway, 2015; Niechwiej-Szwedo et al., 2012).

Hand-eye coordination disorder is closely related to binocular stereo-acuity impairment, visual impairment and reduced contrast sensitivity. For amblyopic children aged 5-6 years, impaired vision-acuity and stereo-acuity are both factors in slower hand movement, but the accuracy of touch is only associated with impaired stereo-acuity. With age (from 7 to 9 years old), there is no difference in the speed of hand movement between normal populations, but the accuracy of touch is still associated with impaired stereo-acuity (Grant et al., 2014; Suttle et al., 2011). Furthermore, the degree of motor skill impairment in adult patients is related to the degree of visual acuity loss (Niechwiej-Szwedo et al., 2014). Multivariate statistics showed that amblyopic eye visual acuity impairment could explain 10% of the variation in touch time and 22% of the variation in touch accuracy, and stereo-acuity impairment explained 23% of the variation in motion control (Niechwiej-Szwedo et al., 2017). It is worth noting that over 50% of these disorder variants cannot be explained by amblyopic eye impairment of visual acuity or stereo-acuity. Future studies are needed to further identify the sources of these disorders.

### 3.2 Egocentric distance perception disorder in action space

**Figure 4:**
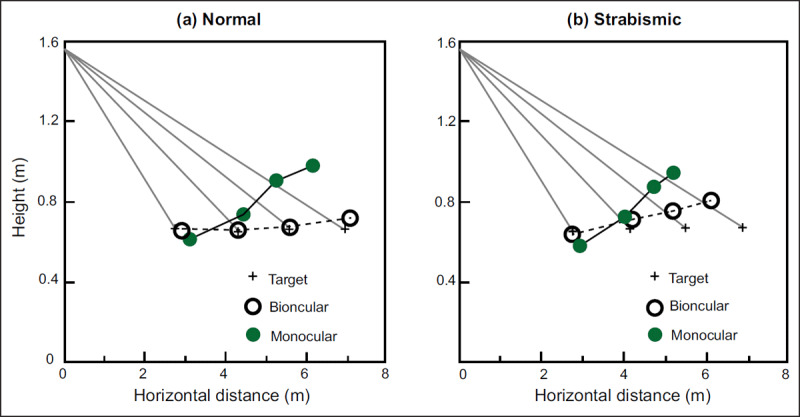
**Amblyopic (a) and normal (b) participants’ judgement of distance in monocular viewing and binocular viewing in the action space**. “+” represents the physical location of the target; the hollow and solid circles represents the location perceived by observers. The zero point on the horizontal axis represents the starting point of the participants, and the zero point on the vertical axis represents the ground. The results in this figure are the distance perception of observers when the target was suspended in the air (0.67 m). Therefore, for either patients with strabismus amblyopia or people with normal vision, egocentric distance perception is better under binocular viewing condition than under monocular viewing condition. In binocular viewing, healthy participants can determine the position of the target more accurately than amblyopes, but not in monocular viewing (Ooi & He, 2015).

**Figure 5:**
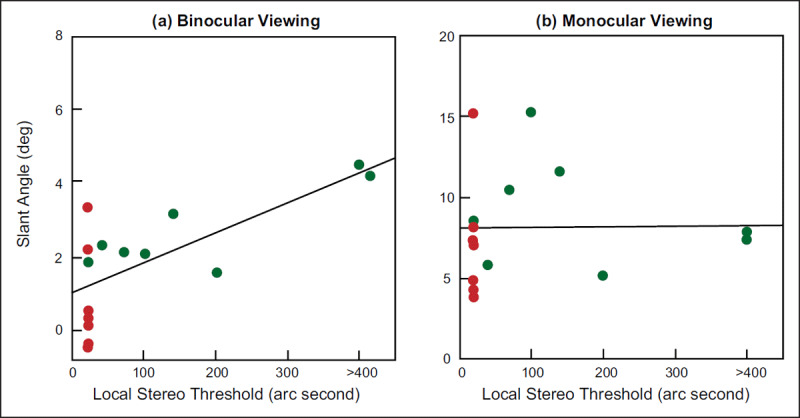
**The relationship between distance perception disorders and stereoscopic vision for healthy people (a) and amblyopes (b) in binocular viewing and monocular viewing**. The solid line shows the degree of fit. There is a strong positive correlation between distance perception defects and stereo perception thresholds in binocular viewing but not in monocular viewing conditions (Ooi & He, 2015).

To the best of our knowledge, only one article directly measured the perception of amblyopes in the action space. Ooi and He (2015) measured the egocentric distance perception of patients with strabismus amblyopia in the range of 2.73 m to 6.93 m (Ooi & He, 2015). Healthy people’s perception of distance is accurate, but the results of distance perception cannot be reported orally (Blake & Sekuler, 2006). For example, we can easily cross the sidewalk, pick up the cup on the table and catch the basketball accurately, but it is difficult to tell how far the screen in front of us is. In this study, a blind walking-gesturing task was used to measure the distance perception in the real world. The researcher placed the target at a distance, and the amblyopes stood in the original location to judge the position of the target without a time limit. After the judgement was completed, the participants were informed to walk quickly to the target with their eyes blindfolded. The distance between the walked position and the starting point was the distance perception of the participants. Ooi and He (2015) examined distance perception under four conditions (2: monocular vs. binocular viewing × 2: target placed on the ground vs. target suspended in the air) simultaneously. When the target was on the ground, both binocular and monocular observations could accurately determine the location of the target, and there was no significant difference between monocular and binocular judgements. This result indicated that regardless of patient status (with strabismus amblyopia or normal), the location of targets on the ground can be accurately judged by monocular cues. However, the distance perception of amblyopic participant was impaired when the target was suspended in the air (0.67 m). In binocular viewing, the normal participants could accurately determine the position of the target (horizontal and vertical positions), but the amblyopes’ judgement of the distant target (5.51 m and 6.93 m) became inaccurate (hollow symbol in ***Figure 4***). Specifically, compared with the actual position of the target, strabismic patients in the binocular viewing condition thought that the distant target was closer to themselves, resulting in more serious space compression. For example, the distance perceived by strabismic participants was 6.2 m when the target was placed at 6.93 m, as illustrated by the cross symbol in ***Figure 4b***. However, normal people have a relatively small bias illustrated by the hollow circle in ***Figure 4a***. By analysing the correlation between stereo perception based on binocular disparity and distance perception disorder, it was found that there was a strong positive correlation between distance perception defect and the stereo perception threshold in binocular viewing (*r^2^* = 0.478, *p <* 0.005), and the poorer the stereoscopic vision was, the poorer the distance perception (***Figure 5a***). This study provides the first evidence for amblyopes judging distance in the action space and clearly proves that there is distance perception disorder in strabismus amblyopic people under binocular conditions.

In monocular viewing, both healthy and strabismic participants exhibited space compression in egocentric distance perception (the horizontal distance of solid symbols in ***Figure 4*** was significantly shorter than that of the “+” symbol), and there was no significant difference between the two groups. The degree of space compression was not correlated with the participants’ stereoscopic vision (*r^2^* = 0.002, *p* = 0.963) (***Figure 5b***). The results suggest that the distance perception of amblyopes based on monocular cues is the same as that of healthy participants. Although the validity of this conclusion is beyond doubt, it is noteworthy that the “monocular condition” in this study was manipulated in the following way: “*An opaque eye patch was used to occlude the observer’s nondominant eye in the monocular condition*” (Ooi & He, 2015). In other words, both groups used the dominant eye to judge distance while occluding the nondominant eye. For amblyopes, the dominant eye is the strong eye; that is, Ooi and He (2015) explored the strong (or healthy) eye of patients with strabismus amblyopia, not the amblyopic eye. Therefore, it remains unclear whether there are disorders of distance perception in amblyopic eyes.

## 4 Future Research Trends

In conclusion, there are disorders in egocentric distance perception in both the personal space and the action space for amblyopes. Specifically, these disorders include decreases in hand-eye coordination ability and target location ability in the range of 2 m to 30 m. However, the disorder of egocentric distance perception with amblyopia is poorly understood. We believe that there are still three questions that urgently need to be addressed. First, the rule of egocentric distance perception in all three spaces is not sufficiently comprehensive. Second, the egocentric distance perception of moving objects in amblyopes remains to be explored. Third, comparative research on the three subtypes of amblyopia is still insufficient. Fourth, study the perception of distance should be conducted under another theoretical framework. Fifth, we should explore the mechanisms of amblyopia by Virtual Reality.

### 4.1 Egocentric distance perception in the three spaces of amblyopia

Researchers have investigated the performance of egocentric distance perception in the personal space and the action space; however, there are still some crucial problems to be urgently addressed. First, is there a disturbance of perception to distance perception in the action space under single-amblyopic eye viewing? Second, egocentric distance perception in the vista space is intimately related to individual planning, navigation, throwing and other abilities, but no research has described the performance of egocentric distance perception of amblyopes in the vista space. Theoretically, people rely on monocular depth cues to judge distance under monocular viewing, which include static and dynamic cues (Howard, 2012). Dynamic cues include optical flow, motion parallax, accumulation and reduction, while static cues include linear perspective, texture perspective, occlusion, transparency, shadows, blurring, and lens accommodation. Visual impairment of amblyopia may affect the perception of these monocular cues. For example, a decrease in sensitivity to high spatial frequency blurs the perception of distant detail cues, which directly affects the linear perspective and texture perspective. Tarampi et al. (2010) used a theatrical lighting gel to simulate monocular vision and contrast sensitivity impairment. The results showed that the status of impairment had no obvious effect on static distance perception (the distance from the target to themselves) within 6 m (Tarampi et al., 2010). However, Rand et al. (2018) discovered by using the same method that the simulated vision loss would affect the distance estimation in the range of 10 m to 30 m; that is, with eyes open and walking for a while, the participants had a feeling of walking farther (Rand et al., 2018). Despite the above hypothesis, the existence of these disorders still needs to be proven with data from real amblyopic participants.

### 4.2 Egocentric distance perception of moving objects in amblyopes

In addition to locating stationary objects, it is important to consider the dynamic location of moving objects. Previous studies have shown that compared with that under binocular viewing, the ability to catch moving objects decreases under monocular viewing, namely, the decrease in egocentric distance perception of moving objects (Lenoir, 2007; Mazyn et al., 2004). People with stereoscopic impairment show safer driving behaviours, such as early braking and slow driving (Grant & Moseley, 2011). However, the probability of traffic accidents in these patients was significantly higher than that in normal people (Maag et al., 1997). Owsley and McGwin reported that there was a weak correlation between visual impairment and accident rate, while peripheral vision decrease had a high correlation with accident rate (Owsley & McGwin, 1999, 2010). The stereoscopic vision, basic visual function and peripheral vision of amblyopes are typically impaired, which implies that amblyopes’ perception of moving objects may also be worse than that of normal people. It is noteworthy that although the above studies support that amblyopes may suffer from driving behavioural disorder, no studies have explored amblyopes’ distance perception of moving objects.

### 4.3 Egocentric distance perception of amblyopic subtypes

Commonly, patients with amblyopia are classified as strabismic amblyopes, anisometropia amblyopes or strabismic-anisometropia amblyopes (McKee et al., 2003). Strabismic amblyopes are amblyopia patients who have strabismus or had strabismus previously. Anisometropia amblyopes are amblyopia patients whose binocular diopters are significantly different (spherical aberration ≥ 1.5 D, columnar aberration ≥ 1.0 D) (American Academy of Ophthalmology et al., 2018). According to an epidemiological survey, strabismic and anisometropia amblyopes each account for approximately 40% and strabismic-anisometropia amblyopes account for approximately 20% of all amblyopic patients (Birch, 2013).

There are remarkable differences between strabismic amblyopes and anisometropia amblyopes. Hess et al. (1980) found that the range of contrast sensitivity impairment was different between the two groups of patients (Hess et al., 1980). Visual impairment in patients with strabismic amblyopes was limited to the central vision (within 5°), while contrast sensitivity impairment occurred in the whole visual field of anisometropia amblyopes. Furthermore, a survey of 427 amblyopes showed that the amblyopes could be divided into four categories according to visual acuity loss and contrast sensitivity loss. Specifically, there were no impairments in visual acuity or contrast sensitivity in normal participants. For strabismic amblyopes, visual acuity is moderately lost, but the contrast sensitivity is the same as that of normal participants. Third, visual acuity of anisometropia amblyopes is moderately lost, but with worse contrast sensitivity than that of normal participants. Fourth, strabismic-anisometropia amblyopes (i.e., mixed amblyopes) suffer from severe visual loss but normal or slightly poor contrast sensitivity (McKee et al., 2003). To this end, although studies have explored the subtype differences of amblyopia, it remains unknown whether and how these differences are related to distance perception. Therefore, researchers need to further clarify the effect of amblyopia types on egocentric distance perception.

### 4.4 Egocentric distance perception in another theoretical framework

To the best of our knowledge, most current researchers explore disorders in egocentric perception in the theoretical framework of Cutting. However, the theory of Cutting is based on the main use of external cues, which is not the necessary classification method used by people for spatial perception. If Cutting’s classification is incorrect, then we may mistakenly attribute some of the defects in the distant space to the close space. Therefore, we need to pay attention to the differences in these classification methods and study the perception of distance between objects under these theoretical frameworks.

According to Previc (1998), we can distinguish three different systems: an action-extrapersonal (for postural control and locomotion) system, which predominantly involves brain areas located in the dorsolateral and dorsomedial cortices (Previc, 1998). The focal-extrapersonal (for visual scanning) and ambient-extrapersonal (for navigation and orientation control) systems predominantly involve brain areas located in the ventrolateral and ventromedial cortices. Another model divided the extrapersonal space into three portions (Grüsser, 1983): (a) a near extrapersonal space (NES) in a zone from 1 to 6/8 metres, (b) a distant extrapersonal space (DES) in a zone between 8 and 30 metres, and (c) the visual background, which extends above 30 metres. The near extrapersonal space (NES) threshold individuated by Grusser (1983) largely overlaps with the threshold identified by Thompson (1983) during a blind walking task (i.e., walking in a certain direction without visual feedback) (Thomson, 1983). The space from 1 to 8 metres may be called the near extrapersonal space (Grüsser, 1983), which is the space where we walk, fight or flee, have social interactions, and see a car approaching or not. Future research on distance perception should consider the role of the body and include age, weight, height, metabolic resources, and gender as covariates.

### 4.5 Virtual reality use in mimicking amblyopia

It is worth noting that even if we clearly analyse the distance perception symptoms of amblyopia patients, this is only the starting point of the investigation. To improve the quality of life of patients with amblyopia, we need to identify the causes of distance perception disorders. Behavioural research is traditionally carried out in a real environment. Thus, it is difficult to manipulate the experimental scenes. It is also difficult for researchers to find the causes of disorder in distance perception. VR is a technology that can construct virtual scenes according to the needs of researchers. Recently, this technology has begun to be applied to amblyopia research (Bao et al., 2018; Bao & Engel, 2019; Dong et al., 2014). However, researchers use this technique to treat amblyopia more than to explore the impairment mechanisms of amblyopia.

From the embodied perspective, the distance in the extrapersonal space is scaled as a function of our action possibilities or opportunities, the related energetic costs and the presence of another individual in the scene (Fini et al., 2014, 2015; Proffitt, 2006b; Proffitt et al., 2003). We consider the virtual reality technique to be better for investigating distance perception in patients with different visual deficits and for testing allocentric distance perception in the presence or absence of other people within the scene.

## 5 Conclusion

Egocentric distance perception plays an important role in patients’ quality of life. However, the mechanism of egocentric distance perception disorder is poorly understood. Hand-eye coordination disorder in the personal space and location disorder of stationary objects in the action space have been studied. In the future, researchers should explore the differences among the three subtypes of amblyopes and study the cognitive mechanism of amblyopes’ egocentric distance perception on the basis of systematically examining the disorder of egocentric distance perception in amblyopes of stationary and moving objects in the three spaces.

According to the Sixth National Census and the incidence of amblyopia, there are approximately 17 million amblyopic patients in China (Population Census Office under the State Council & Department of Population and Employment Statistics National Bureau of Statistics, 2012). The cure rate of amblyopia is only 60% to 70%, which means that the quality of life of nearly 5.1 million to 6.8 million amblyopic patients is affected in China alone. Meanwhile, the United Nations expects the world’s population to reach 8.6 billion by 2030 (United Nations et al., 2017). At that time, there will be approximately 150 million individuals with amblyopia in the world. Further research should clarify the symptoms, mechanisms and corrective measures of egocentric distance perception disorder of amblyopes as soon as possible and strive to improve the quality of life of diseased patients.
